# Withaferin A Alone and in Combination with Cisplatin Suppresses Growth and Metastasis of Ovarian Cancer by Targeting Putative Cancer Stem Cells

**DOI:** 10.1371/journal.pone.0107596

**Published:** 2014-09-29

**Authors:** Sham S. Kakar, Mariusz Z. Ratajczak, Karen S. Powell, Mana Moghadamfalahi, Donald M. Miller, Surinder K. Batra, Sanjay K. Singh

**Affiliations:** 1 Department of Physiology and Biophysics, University of Louisville, Louisville, Kentucky, United States of America; 2 James Graham Brown Cancer Center, University of Louisville, Louisville, Kentucky, United States of America; 3 Department of Medicine, University of Louisville, Louisville, Kentucky, United States of America; 4 Research Resources Center, University of Louisville, Louisville, Kentucky, United States of America; 5 Department of Pathology, University of Louisville, Louisville, Kentucky, United States of America; 6 Department of Biochemistry and Molecular Biology, Eppley Institute for Research in Cancer and Allied Diseases, University of Nebraska Medical Center, Omaha, Nebraska, United States of America; Florida International University, United States of America

## Abstract

Currently, the treatment for ovarian cancer entails cytoreductive surgery followed by chemotherapy, mainly, carboplatin combined with paclitaxel. Although this regimen is initially effective in a high percentage of cases, unfortunately within few months of initial treatment, tumor relapse occurs because of platinum-resistance. This is attributed to chemo-resistance of cancer stem cells (CSCs). Herein we show for the first time that withaferin A (WFA), a bioactive compound isolated from the plant Withania somnifera, when used alone or in combination with cisplatin (CIS) targets putative CSCs. Treatment of nude mice bearing orthotopic ovarian tumors generated by injecting human ovarian epithelial cancer cell line (A2780) with WFA and cisplatin (WFA) alone or in combination resulted in a 70 to 80% reduction in tumor growth and complete inhibition of metastasis to other organs compared to untreated controls. Histochemical and Western blot analysis of the tumors revealed that inclusion of WFA (2 mg/kg) resulted in a highly significant elimination of cells expressing CSC markers - CD44, CD24, CD34, CD117 and Oct4 and downregulation of Notch1, Hes1 and Hey1 genes. In contrast treatment of mice with CIS alone (6 mg/kg) had opposite effect on those cells. Increase in cells expressing CSC markers and Notch1 signaling pathway in tumors exposed to CIS may explain recurrence of cancer in patients treated with carboplatin and paclitaxel. Since, WFA alone or in combination with CIS eliminates putative CSCs, we conclude that WFA in combination with CIS may present more efficacious therapy for ovarian cancer.

## Introduction

Epithelial ovarian cancer (EOC) remains the leading cause of death in women among gynecologic cancers and is the 5th highest cause of cancer-related deaths in women in the United States [1], [2]. The majority of ovarian cancers are diagnosed at advanced stage due to the mainly non-specific symptoms. Currently, the treatment for ovarian cancer entails cytoreductive surgery followed by chemotherapy, employing mainly platinum/taxane combination [3]. Although this regimen is initially effective in a high percentage of cases (70 to 80%), unfortunately 70% of women develop recurrent cancer within few months of initial treatment as a result of platinum-resistance [4], [5]. In addition, cisplatin (CIS) is associated with multiple severe side effects such as nausea, vomiting, myelosuppression, hepatotoxicity, neurotoxicity, nephrotoxicity and ototoxicity [4], [6]–[9]. Therefore, need for new treatment options that target cancer cells and in particular putative cancer stem cells is mandatory either at first-line setting or even more at the first- and second-line management of recurrent ovarian cancer.

In our previous studies [10], we showed for a first time that withaferin A (WFA), a bioactive compound isolated from the plant Withania somnifera, when used alone or in combination with CIS had a time- and dose-dependent synergistic effect on inhibition of cell proliferation and induction of cell death, thus reducing required dosage of cisplatin. We also showed that while WFA achieves its antitumor effect through generation of ROS leading to DNA damage, CIS achieves its effects though direct binding to DNA causing the formation of DNA adducts. Combination treatment also resulted in a significant enhancement of reactive oxygen species (ROS) production and DNA damage.

WFA has been a part of Indian traditional medicine for centuries. It is available in US over-the-counter as a dietary supplement and is known to treat various disorders due to its anti-inflammatory [11], [12], anti-bacterial [13], and cardio protective properties [14]. In recent years, WFA has been suggested as a potential anti-cancer compound shown to prevent tumor growth, angiogenesis, and metastasis [15], [16] in various types of cancer [17]–[26]. Mechanisms by which WFA attains its anticancer activity include inactivation of Akt and NF-κB [27] to achieve apoptosis, decrease in pro-survival protein Bcl-2 [28], G2/M cell cycle arrest [29], [30], generation of reactive oxygen species (ROS) [31], [32], induction of Par-4 [17], activation of caspase 3 and 9 activities, DNA damage [10], inhibition of HSP90 [20], regulation of FOXO3a and Bim [15] inhibition of Notch-1 [33] and down regulation of expression of HPV E6 and E7 oncoproteins [19].

Development of drug resistance and recurrence of ovarian cancer has been a major clinical problem. A number of mechanisms that induce drug resistance have been proposed. Over the last several years, there has been increasing evidence that “cancer stem cells (CSCs)”, are the most important trigger of tumor progression, chemo-resistance and relapse after initial treatment [34], [35]. First evidence for the existence of cancer stem cells came in the year 1997, with the identification of leukemia stem cells [36], [37]. In the year 2003, Al-Hajj et al. [38] experimentally demonstrated the hierarchical stem cell origin in breast cancer. However, until recently the existence of putative cancer stem cells within solid tumors had remained controversial [39]. In recent studies using murine models for brain, skin and intestinal tumors, three independent groups have provided convincing evidence for the existence of CSCs in tumors and their role in tumor expansion [40]–[42]. Accordingly, CSCs within tumor mass undergo self-renewal and give rise to heterogeneous cancer lineages that comprise tumor tissue. CSCs purified accordingly to some surface markers are able to form tumors when injected into nude mice [36], [43], [44]. Since, ovarian cancer is very heterogeneous; different cell surface markers have been reported for putative ovarian CSCs. Most commonly reported include CD24, CD34, CD44, CD133, CD117, ALDH1, Oct4, MyD88 and EpCAM [45]–[53]. Since, CSCs are considered to be major players responsible for developing drug resistance and hence leading to cancer recurrence [52], [53], targeting CSCs and inhibiting their self-renewal will lead to reduction of cancer growth [33].

In our current study, we show for the first time that WFA alone or in combination with CIS if employed to treat mice bearing human orthotopic ovarian tumors not only suppresses tumor growth but targets cells expressing CSC markers as well as inhibits Notch1 and its downstream signaling genes (Hes1 and Hey1) that have been reported to play a crucial role in self-renewal and maintenance of CSCs (33).

## Material and Methods

### Cell line and cell culture

Ovarian epithelial cancer cell line A2780 was initially obtained from Denise Connolly (Fox Chase Cancer Center) and was maintained in RPMI 1640 medium containing insulin and supplemented with penicillin/streptomycin (100 IU/ml and 100 µg/ml) and 10% fetal bovine serum (FCS) (Hyclone, Atlanta, GA) as described previously [10].

### Cell migration Boyden chamber assays

Cell migration in vitro was assayed by determining the ability of cells to migrate through a synthetic basement membrane. The procedure used was as described previously [54]. Briefly, polycarbonate filters (8 µM) were placed in modified Boyden chamber. A2780 cells in log phase were trypsinized and plated in 6 wells plates. After 24 h of plating, cells were treated with WFA and CIS both alone and in combination as described previously [10]. After 24 h of treatment, cells were trypsinized and suspended in serum free medium. A total of 2×10^5^ cells were transferred to the top chamber. The medium containing 5% FBS was added to the lower chamber. The cells were incubated at 37°C for 24 h and allowed to migrate through the membrane. Non-migrated cells were removed with a clean cotton swab. Migrated cells on other side of the membrane were stained with crystal violet and counted in three different fields under Olympus microscope. The experiments were repeated for three times. The values represented are the mean ± SEM of three independent experiments.

### Generation of orthotopic ovarian tumors in nude mice and treatment with WFA and CIS both alone and in combination

Orthotopic ovarian tumors were generated by injecting ovarian cancer cell line A2780 directly into ovary as described by Nunez Cruz et al. [55]. Briefly, A2780 (1×10^6^) cells were directly injected into left ovary of 5 to 6 weeks old nu/nu female mice (Jackson Laboratory) under aseptic conditions and under light anesthesia. After 10 days of post-cell injection, mice were treated with 1) vehicle control (10% DMSO and 90% glyceryl trioctanoate), 2) WFA 2 mg/kg, 3), CIS 6 mg/kg, and 4) WFA 2 mg/kg plus CIS 6 mg/kg. Five animals randomly were included in each group. CIS in saline was injected i.p. once a week, whereas WFA was injected i.p. every other day. After 4 weeks of treatment, animals were sacrificed; tumor and other tissues such as un-injected ovary, lung, kidney, liver, adrenal and heart were collected from each mouse. Tumors were weighted at the time of collection. The tumors and other tissues were divided into two parts, one part was snap frozen, and second part was fixed in 10% buffered formalin. The animals' experiments were approved by the University of Louisville, Institutional Animal Care and Use Committee (IACUC) (protocol # 12063).

Formalin fixed tumor and tissues were processed and embedded in paraffin using standard protocols as described previously [56]. Five µM thick sections of the embedded tumors and tissues were prepared and stained with Hematoxylin and Eosin (H&E). Sections in triplicates were examined under microscope and photographed. Histopatholoigcal analysis of sections was performed by a trained pathologist Dr. Mana Moghadamfalahi.

### Immunohistochemistry

Formalin fixed paraffin embedded tissues were deparaffinized in xylene and rehydrated in a decreasing graded series of ethanol as described previously [56]. Sections were heated at 95°C in 10 mM sodium citrate (pH 6.0) for 20 min, cooled to room temperature and then rinsed in PBS. Sections were incubated with 0.3% hydrogen peroxide in methanol for 10 min at room temperature to quench endogenous peroxidase followed by two rinses in PBS (5 min each), and were blocked with normal goat serum using reagents from ABC kit from Vector Laboratories for 60 min at room temperature following the instructions from the supplier. The blocking solution was removed by draining and sections were incubated with specific antibody with appropriate dilution according to instructions from the suppliers at 4°C for overnight in a humidified chamber. The antibodies for CD24 (cat # SAB14202713), CD44 (cat # SAB1405590), CD117 (cat # SAB4300489) and Oct4 (cat # P0873) were obtained from Sigma-Aldrich, and antibody for CD34 (cat # sc-19587) was obtained from Santa Cruz Biotechnology. After rinsing the sections three times (5 min each) with PBS, sections were incubated with biotinylated anti-rabbit (for polyclonal antibodies) or anti-mouse (for monoclonal antibodies) from the ABC kits (Vector Laboratories) at room temperature for 45 min followed by incubation with streptavidin. After three rinses (5 min each) with PBS, sections were incubated with 3,3′-diaminobenzidine (DAB, Sigma) to develop color. The sections were examined under Nikon Elipse E400 microscope and photographed.

### Protein isolation and western blot analysis

A2780 cells were plated into 6 well plates. After 24 h of plating, cells were treated with WFA and CIS both alone and in combination as described previously (10). After 48 h of treatment, cells were lysed in chilled lysis buffer [50 mM Tris-HCl (pH 7.5), 150 mM NaCl, 0.1% NP-40, 1 mM Na_3_VO_4_, and 1 mM NaF) supplemented with Complete Mini Protease Inhibitor tablet (Roche Molecular Biochemicals, Indianapolis, IN). To prepare extract from normal and tumor tissues, tissues were suspended in lysis buffer and homogenized on ice using Polytron homogenizer followed by centrifugation at 10,000 rpm for 5 min. The supernatants were collected and protein concentration in each sample was determined using Bradford method (BioRad Laboratories) according to supplier's instructions. Forty µg of protein from each sample was fractionated on SDS-polyacrylamide gels and transferred to nitrocellulose membranes as described previously [56]. Blocking of nonspecific proteins was performed by incubation of the membranes with 5% nonfat dry milk in Tris buffered saline Tween-20 (TBST) for 1 h at room temperature. The membranes were incubated with specific antibody with appropriate dilution as suggested by the suppliers. Antibody for Notch 1 (cat # N6786), Hey1 (cat # SAB1404975) and β-actin (cat # A3854) were obtained from Sigma-Aldrich, and antibody for Hes1 (cat # sc-165996) was obtained from Santa Cruz Biotechnology. The membranes were washed three times (5 min each) with TBST, followed by incubation with horseradish peroxidase conjugated secondary antibody (1∶5,000 dilution) in TBST. The membranes were rinsed three times (5 min each) with TBST and the immuno-reactive bands were visualized by enhanced chemiluminescence. Membranes were stripped off for 10 min with methanol containing 3% H_2_O_2_ and probed with β-actin antibody in order to serve as an internal control.

### Statistical analysis

Statistical comparison of data was carried out by the student's t test (for single comparison). Probability of p<0.05 determined from the two-sided test was considered significant. The statistical analysis was carried out by using SPSS 10.0 software.

## Results

### WFA/CIS combination inhibits cell migration in vitro

**Figure 1:**
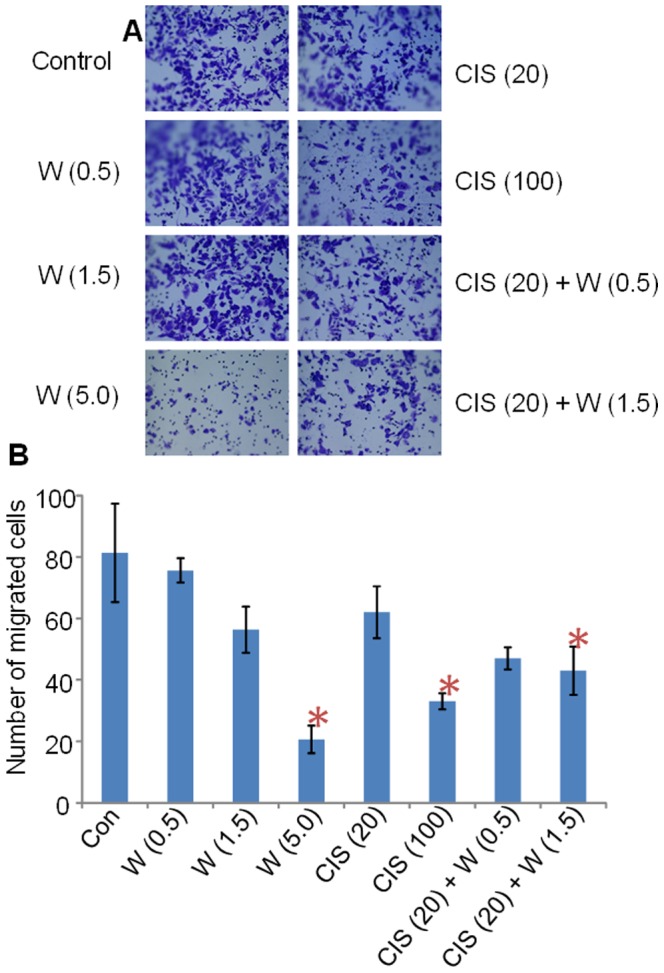
Effect of WFA and CIS both alone and in combination on cell migration. A2780 cells were treated with different concentration of WFA and CIS both alone and in combination for 48 h. The cells were trypsinized and subjected for cell migration using Boyden chamber. Cells were stained with crystal violet and photographed (A). The stained cells were counted under microscope using three different areas; values shown are mean ± SD of three independent experiments. * Represents significant compared to control at p≤0.05 (B). Con  =  control, W  =  WFA. Values shown in parenthesis are µM.

Various steps are involved in tumor progression and metastasis including detachment of tumor cells from the primary tumor site, transmigration into lymph- or blood vessels, attachment to endothelium at distant sites of metastasis followed by seeding into new location and subsequent expansion. To examine the effect of WFA and CIS on A2780 cell migration, we treated the A2780 cells with WFA and CIS both alone and in combination for 48 h. As shown in Fig. 1, by employing Boyden chambers we noticed that the treatment of cells with WFA or CIS alone inhibited cell migration in a dose-dependent manner as compared to untreated control cells. While treatment of cells with 20 µM CIS inhibited cell migration, addition of WFA (0.5 µM or 1.5 µM) to CIS resulted in enhanced inhibition of cell migration, suggesting that WFA combined with CIS is more effective than each agent employed.

### WFA/CIS combination suppresses tumor growth and metastasis in nude mice

**Figure 2:**
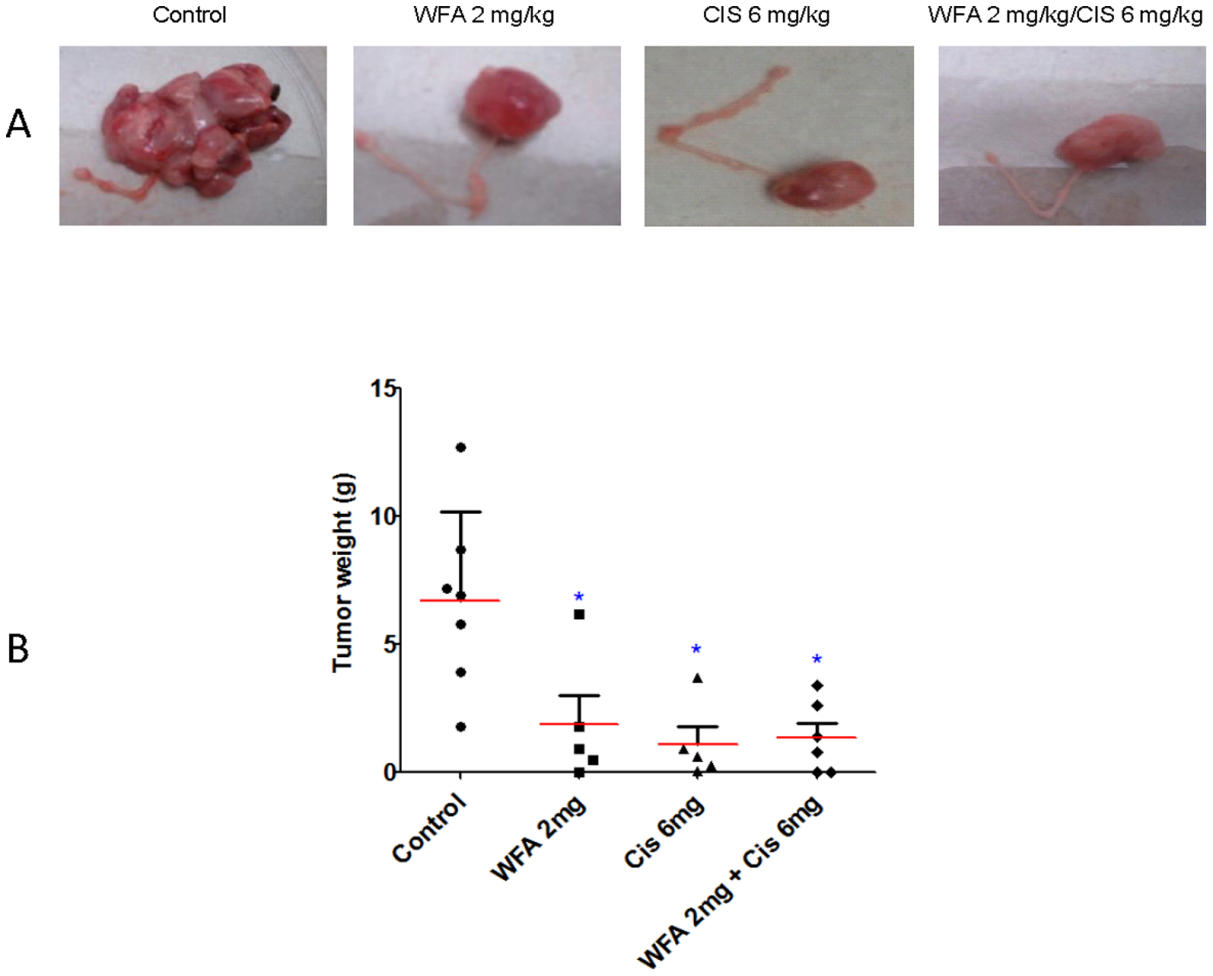
Effect of WFA and CIS treatment on tumor growth. A: 1×10^6^ A2780 cells were injected into female mouse ovary. After 10 days of post-injection, mice were treated with WFA and CIS both alone or in combination for four weeks. Mice were sacrificed; tumors were excised out, photographed and weighted. Tumors shown are representative from each group. B: Tumors weight was plotted from each group. Horizontal line represents median weight of each group. Treated group showed significantly lower weight than untreated mice. Results are mean (red line) and ± SD (vertical bar). * Represents significant compared to control at p≤0.05.

In our *in vitro* studies, we showed that treatment of CIS-sensitive cell lines (A2780 and CaOV3) as well as CIS-resistance cell line (A2780/CP70) with WFA and CIS both alone and in combination inhibited cell proliferation in a time- and dose-dependent manner and induced cell apoptosis and DNA damage. Moreover, the combined effect of WFA and CIS was synergistic [10]. To assess the efficacy of WFA/CIS combination on tumor growth and metastasis in vivo, we tested the effect of WFA and CIS both alone and in combination on tumor growth and metastasis in nude mice bearing inoculated orthotopic human ovarian tumors. Murine orthotopic tumors were established by injecting A2780 cells directly into left ovary of 5 to 6 week old nu/nu female mice. Beginning from day 10 after inoculation of tumor cells, animals were treated with WFA and CIS both alone and in combination as detailed in Materials and Methods section. After 4 weeks of treatment, animals were sacrificed. We noticed that the control mock-treated animals developed highly vascularized and large tumors (Fig. 2). At the same time 4 out of 5 WFA (2 mg/kg) alone-treated animals developed tumors that were significantly smaller in size. Similarly, 3 out of 5 animals treated with CIS (6 mg/kg) developed tumors that were significantly smaller in size as compared to mock-treated controls. Moreover, treatment of animals with WFA (2 mg/kg) in combination with CIS (6 mg/kg) resulted in 70 to 80% reduction in tumor weight compared to untreated control animals (Fig. 2) and out of 5 mice, only three mice developed tumors. No significant differences in tumor weight were observed in mice treated with WFA and CIS alone or in combination (Fig 2).

**Figure 3:**
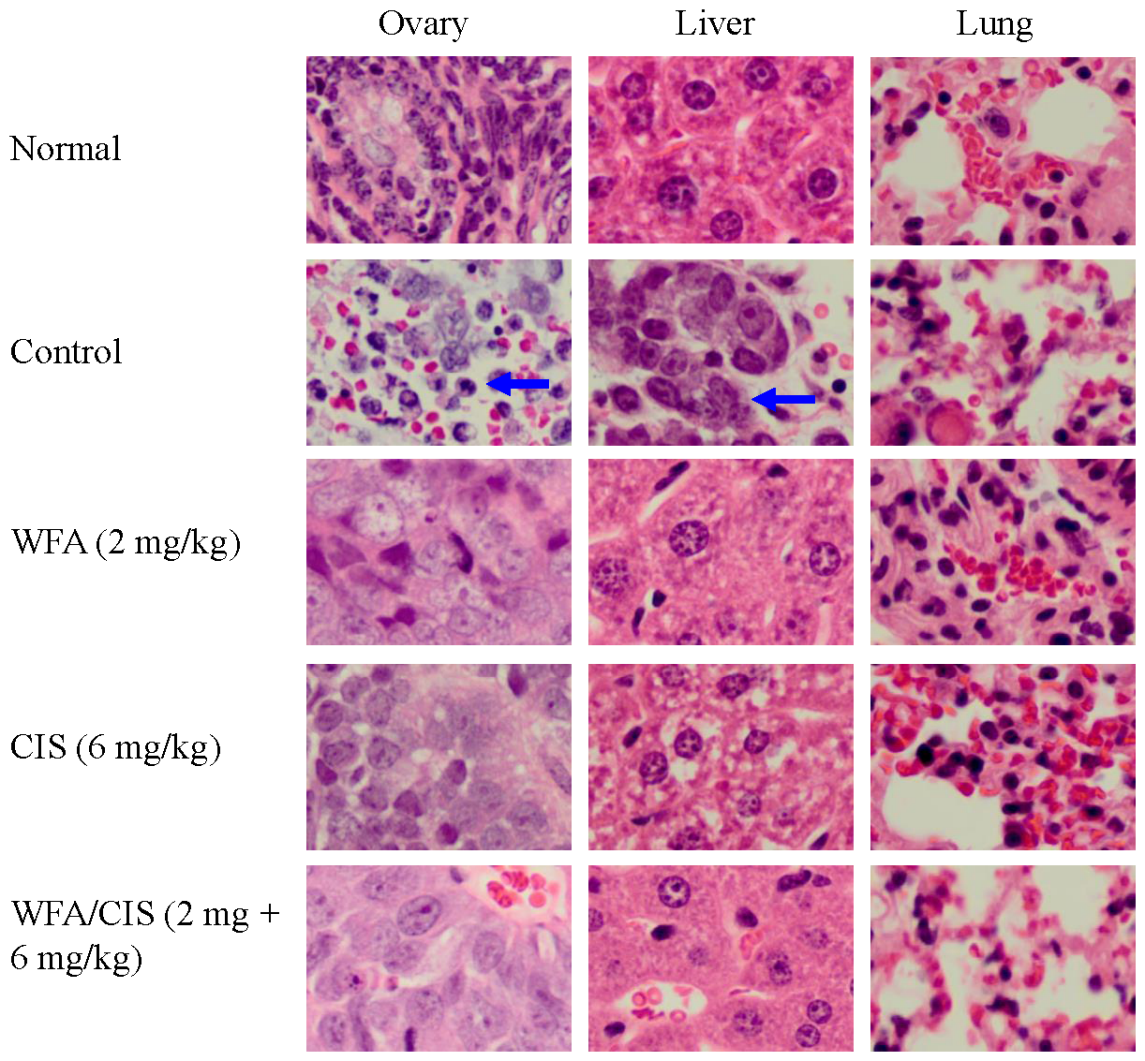
Effect of WFA and CIS both alone and in combination on tumor metastasis. Mice were treated with WFA and CIS as indicated in Figure 2. Tumors and other tissues sections were stained with H&E and examined by a trained pathologist. Metastasis (shown by arrows) was observed in un-injected ovaries and livers and represent approximate 10% of the cells.

H&E histo-pathological analysis of un-injected opposite ovaries, livers, and lungs showed metastasis to livers and ovaries in mock- treated animals only. Metastatic cells comprised ∼10% of cells in those organs (Fig. 3). In contrast no metastases were observed in WFA and CIS treated groups. These results suggest that combination of low dose of WFA (2 mg/kg) with suboptimal dose of CIS (6 mg/kg) is highly effective in suppressing tumor growth and metastasis of orthotopic ovarian tumors in nude mice. This indicates that it would be possible to reduce therapeutic dose of CIS when combined with WFA in humans to ameliorate side effects associated with high dosage of CIS.

### WFA alone or in combination with CIS eliminates putative cancer stem cells in orthotopic ovarian tumors

**Figure 4:**
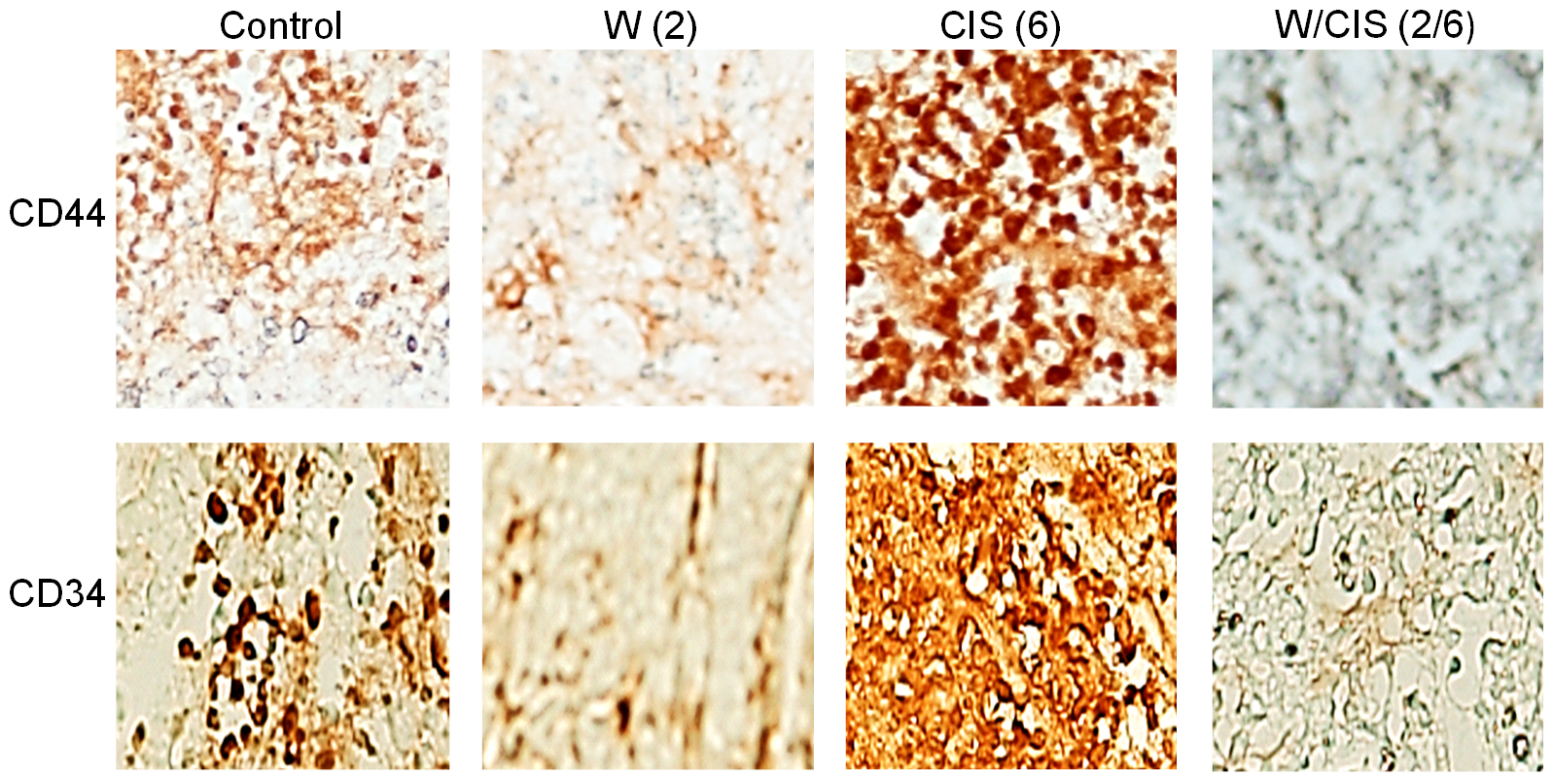
Immunohistochemical analysis of CD44 and CD34 positive cells in tumors collected from mock treated mice (control) and mice treated with WFA and CIS both alone and in combination. The data shown is representative of two independent experiments. W  =  WFA. Values shown in parenthesis are mg/kg.

**Figure 5:**
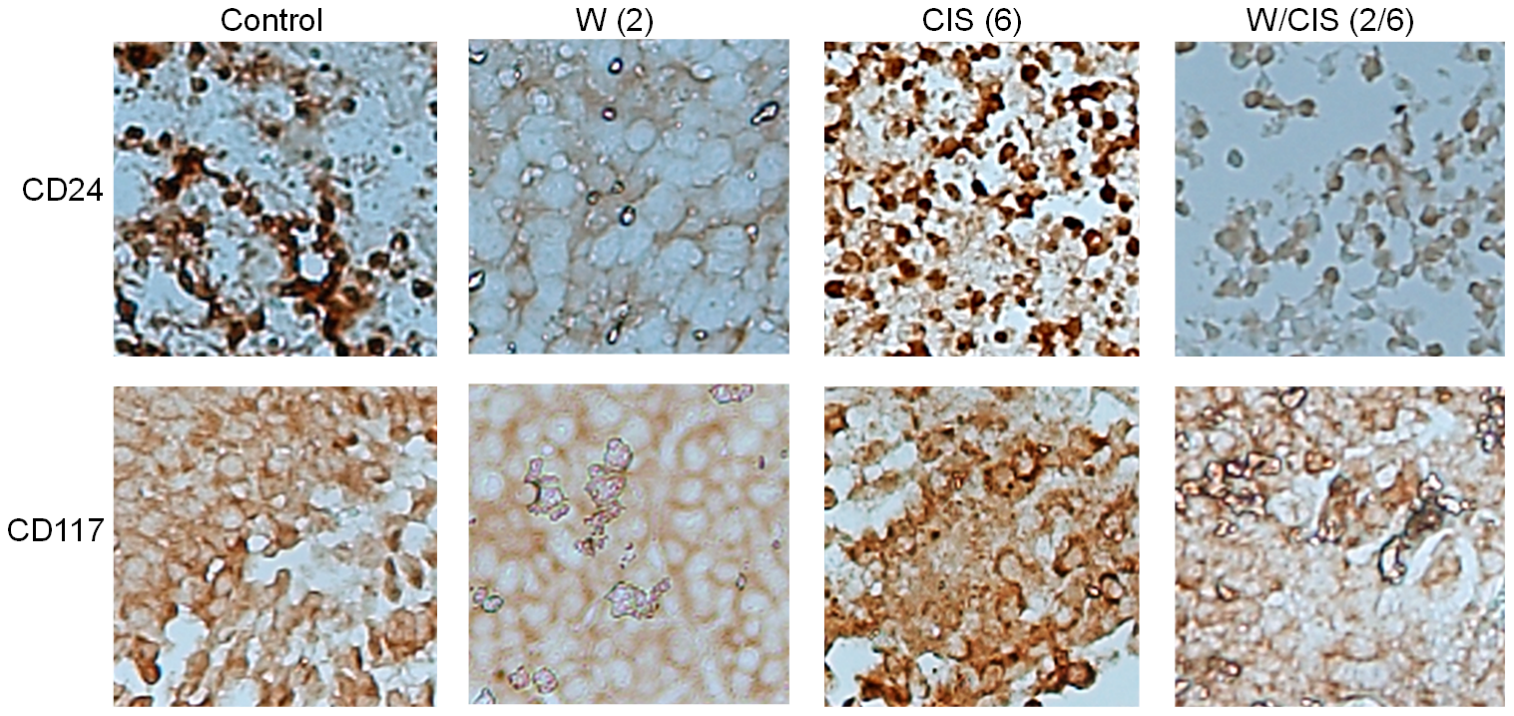
Immunohistochemical analysis of CD24 and CD117 positive cells in tumors collected from mock treated mice (control) and mice treated with WFA and CIS both alone and in combination. The data shown is representative of two independent experiments. W  =  WFA. Values shown in parenthesis are mg/kg.

**Figure 6:**
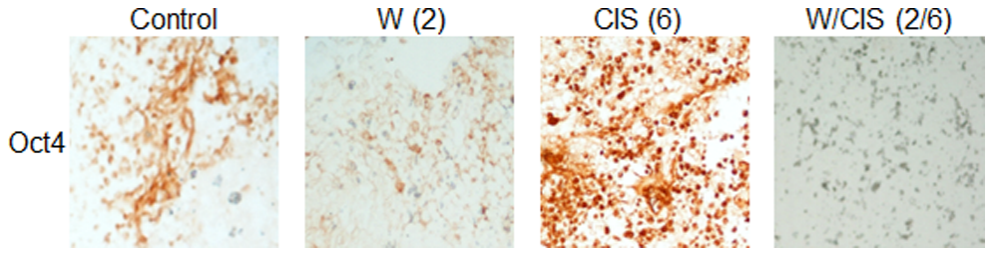
Immunohistochemical analysis of Oct4 positive cells in tumors collected from mock treated mice (control) and mice treated with WFA and CIS both alone and in combination. The data shown is representative of two independent experiments. W  =  WFA. Values shown in parenthesis are mg/kg.

Chemo-resistance and recurrence of ovarian cancer is a major problem and cause of death. In recent years, a concept of CSCs in solid cancers including ovarian cancers has been proposed [57], [58]. CSCs have been reported to be responsible for chemo-resistance, tumor growth and recurrence of cancer after treatment. Putative CSCs have been reported as cancer initiating cells capable to develop tumors when injected into nude mice [57]. To test if WFA when used alone or in combination with CIS targets CSCs, we performed immunohistochemical analysis of the tumors collected from the mock-treated animals and animals treated with WFA and CIS both alone and in combination using the antibodies for markers expressed by putative CSCs including CD44, CD24, CD34, CD117 and Oct4 [56]. As shown in Figs. 4–6, we observed ∼10–20% of cells positive for CD44, CD24, CD34, CD117 and Oct4 in tumors collected from untreated animals. However, treatment of animals with WFA (2 mg/kg) resulted in a highly significant reduction in number of those cells. In contrast, treatment of animals with CIS alone at a dose of 6 mg/kg resulted in significant increase in CD44, CD24, CD34, CD117 and Oct 4 positive cells (60%) (Figs. 4–6). More importantly, treatment of animals with WFA in combination with CIS (6 mg/kg) significantly reduced number of cells expressing CSC markers.

### WFA alone or in combination with CIS down regulates the expression of CSC-related markers

**Figure 7:**
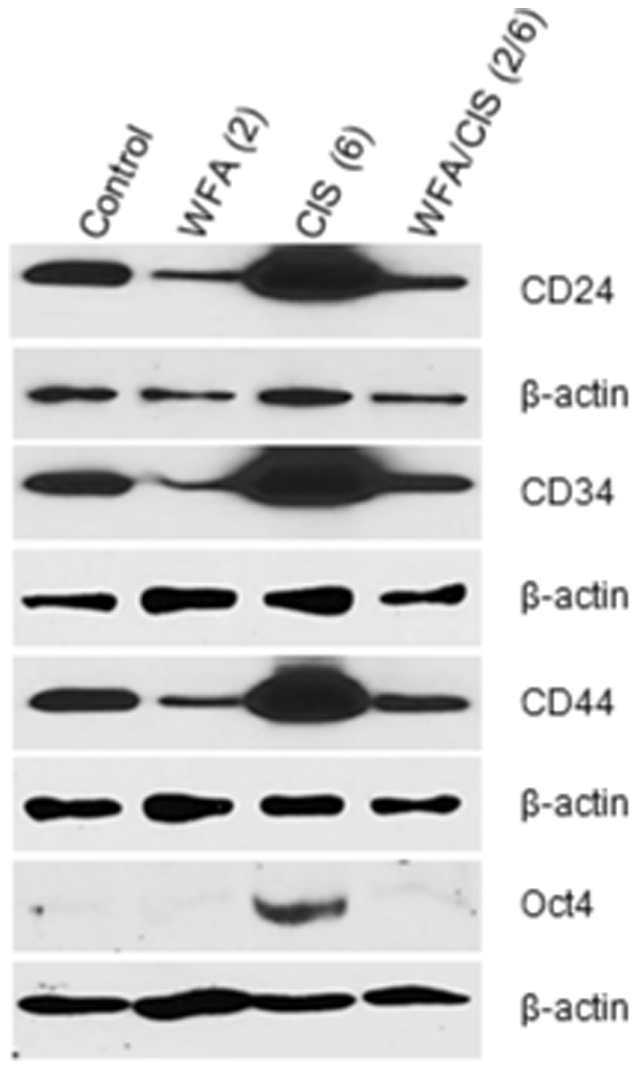
Western lot analysis of CD24, CD34, CD44, and Oct4 proteins from tumors collected from mock treated mice and mice treated with WFA and CIS both alone and in combination. Beta-actin was used as an internal control. The data shown is representative of two independent experiments. Con  =  control, W  =  WFA. Values shown in parenthesis are mg/kg.

To confirm our immuno-histochemical analysis of CD44, CD24, CD34, CD117 and Oct4 positive cells in orthotopic tumors, we performed Western blot analysis of the tumor extracts using specific antibody for markers detected by immuno-histochemical staining. As shown in Fig. 7, expression of CD24, CD34, CD44, and Oct4 antigens was significantly down-regulated in tumors collected from animals treated with WFA alone as compared to tumors from mock-treated animals. In contrast a significant increase in expression of CD24, CD34, CD44 and Oct4 was observed in tumor extracts from animals treated with CIS (6 mg/kg) as compared to mock-treated mice or mice treated with WFA (2 mg/kg) alone. Interestingly, treatment of animals with WFA (2 mg/kg) in combination with CIS (6 mg/kg) resulted in a significant elimination of cells expressing CD44, CD24, CD34 and Oct4 antigens.

Increase in number of cells expressing markers of putative CSCs in tumors collected from animals treated with CIS as analyzed by immuno-staining as well as Western blot analysis suggests that treatment by CIS may increase number of cells expressing these markers and may explain development of chemo-resistance and reoccurrence of ovarian cancer in patients treated with CIS or its derivative such as carboplatin in combination with paclitaxel that are commonly used in chemotherapy. In contrast elimination of cells expressing CSC markers in tumors on treatment with WFA alone or in combination with CIS (6 mg/kg) demonstrates that WFA is highly effective in eliminating cells expressing CSC markers.

### WFA alone or in combination with CIS inhibits Notch 1 and its downstream signaling genes (Hes1 and Hey1)

**Figure 8:**
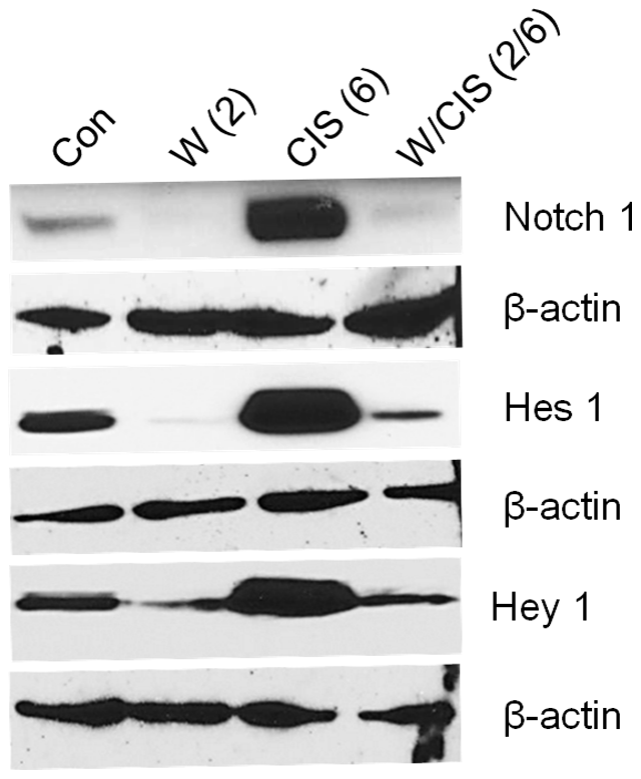
Western blot analysis of Notch1, Hes1 and Hey 1 proteins from tumors collected from mock treated mice and mice treated with WFA and CIS both alone and in combination. Beta-actin was used as an internal control. The data shown is representative of two independent experiments. Con  =  control, W  =  WFA. Values shown in parenthesis are mg/kg.

Self-renewal, drug resistance and differentiation are key characteristics of CSCs. Sonic Hedgehog (Shh), Notch1, Twist1, Snail and Wnt1 signaling transduction pathways play major roles in the self-renewal of these cells [33], [59]–[68]. WFA has been reported to inhibit Notch-1 and downstream signaling genes (Hes1 and Hey1) [33], [68]. Notch 1 signaling pathway is associated with regulation of cell fate at several distinct developmental stages and has been implicated in cancer initiation and progression [63], [69], [70]. In our present study as shown in Fig. 8, we noticed highly significant inhibition of expression of Notch 1 and its downstream signaling genes Hes1 and Hey1 in tumors collected from mice treated with WFA (2 mg/kg) as compared to tumors from control mock-treated animals. In contrast, animals treated with CIS (6 mg/kg) showed a highly significant increase in levels of Notch1, Hes1 and Hey1 genes. What is important, tumors collected from mice treated with WFA (2 mg/kg) in combination with CIS (6 mg/kg) showed significant decreased levels of Notch1 as well as Hes1 and Hey1 proteins (Fig. 8), suggesting downregulation of Notch1 signaling by WFA alone or in combination with CIS leading to elimination of putative CSCs.

## Discussion

The most common first line chemotherapy used for ovarian cancer after cytoreductive surgery is carboplatin in combination with paclitaxel. Initial response rate to this combination is very high (70 to 80%), however within 6 to 20 months after initial treatment tumor relapse and patients become resistance to CIS [71]. Resistance to CIS has been associated with number of mechanisms such as increase in glutathione and metallothionein levels, decrease in drug uptake, increase in DNA repair mechanisms (due to enhanced expression of excision repair genes) and tolerance of the formation of platinum-DNA adducts [72]. Change in status of p53 has also been reported to play important role in sensitivity of CIS [73], [74].

In recent years, several investigators have reported a presence of small population of CSCs in tumor tissues to be responsible for induction of chemo-resistance and recurrence of cancer [75]–[77]. The convincing evidence for the role of CSCs in ovarian cancer was provided by Bapat et al. [45] who showed the presence of CSCs at single cell level in the ascites of an ovarian cancer patient, that could sequentially propagate tumor over several generations. Consistent with this, many other investigators reported the presence of CSCs in ovarian cancer cell lines, patients' ovarian tumors and tumor associated-ascites [57], [76], [77]. As a follow up of these observations CSCs have been isolated based on the presence of some extracellular markers. Most common makers used for ovarian CSCs include CD44, CD24, CD34, CD117 and CD133. CSCs also express ALDH1, Oct4, Myd88 and EpCAM [47], [51], [57], [60], [78], [79]. An increase in number of CSCs in ovarian tumors correlates with a poor prognosis, including shorter overall and disease free survival [80]–[82]. Development of chemo-resistance of ovarian cancer could be explained by enrichment for CSCs [77], [83]–[85]. In a recent study, Abubaker et al. [53] demonstrated using two ovarian cancer cell lines (epithelial OVCA433 and mesenchymal HEY) enrichment for a population of cells with high expression of CSC markers at the protein as well as mRNA levels after treatment with CIS, paclitaxel and the combination of both. In addition, these investigators showed increase in tumorigenic properties of ovarian cancer cells in response to chemotherapy drugs. In the present study, we show somehow in agreement with those studies [53] that the number of CSCs increases in animals bearing orthotopic ovarian tumors treated with CIS at 6 mg/kg. This increase in CSCs population in ovarian tumors of mice with CIS may explain the development of chemo-resistance and reoccurrence of ovarian cancer in patients treated with CIS or its derivative carboplatin employed in combination with paclitaxel.

Increase in number of CSCs in tumors inoculated in nude mice followed by CIS treatment is result of amplification of CSCs present in human cancer cell line A2780. On other hand growing tumor will attract host-derived normal stem cells that will provide stroma and vasculature for expanding tumor tissue. These cells could provide trophic signals for CSCs, and this is currently investigated in our laboratories.

In the past years a great deal of efforts has been devoted to develop drugs that can kill cancer cells as well as CSCs in order to reduce chemo-resistance and recurrence of cancer after treatment. WFA as reported exhibits an inhibitory effect against several different types of cancer cells. However, its effect on CSCs has not been explored so far. In our previous study [10], we demonstrated that WFA when used alone or in combination with CIS inhibits cell proliferation and induce cell death of both CIS-sensitive (A2780 and CaOV3) as well as CIS-resistant (A2780/CP70) cell lines. In our present follow-up study we show that WFA (2 mg/kg) when used alone or in combination with CIS to treat mice bearing orthotopic ovarian tumor reduced tumor growth by 70 to 80% and prevented metastasis to other organs. In addition, treatment of mice bearing orthotopic ovarian tumors with WFA alone or WFA + CIS eliminated cells that express CSC markers. (CD44, CD24, CD34, CD117 and Oct4). In contrast the number of these cells as mentioned above increased in our hands after treatment by CIS alone. Thus, our results clearly demonstrate that combination of low dose of WFA (2 mg/kg) with suboptimal dose of CIS (6 mg/kg) is highly effective in suppressing the tumor growth and elimination of putative CSCs “expanded” by CIS treatment. Since, therapeutic dose of CIS is 8 mg/kg [19], WFA in combination with CIS has potential to be highly effective and efficacious therapy for ovarian cancer and may ameliorate CIS-therapy related side effects.

Self-renewal, drug resistance and differentiation are key characteristics of CSCs and several developmental pathways such as Sonic Hedgehog (Shh), Notch, Wnt and TGFβ, Twist, and Snail which have been shown to be crucial in these processes [33], [59]–[67], [86]. WFA has been reported to inhibit Notch1 and downstream signaling genes (Hes1 and Hey1) [43], [77] that have been implicated in cancer initiation and progression [63], [69], [70].

In our present study, we show for a first time highly significant inhibition of Notch1 and its downstream signaling proteins Hes1 and Hey1 in tumors collected from animals treated with WFA (2 mg/kg) as compared to tumors from mock-treated animals. In contrast, animals treated with CIS (6 mg/kg) alone showed a significant increase in levels of Notch1, Hes1 and Hey1 genes which is consistent with the increase in number of CSCs, suggesting an important role of Notch1 transduction pathway in amplification of those cells. More importantly, treatment of animals with WFA (2 mg/kg) + CIS (6 mg/kg) prevented increase of Notch1, Hes1 and Hey1 expression, suggests that such combined therapy ameliorates unwanted effect of CIS treatment alone and unwanted expansion of CSCs. Thus, treatment of patients that have become resistance to CIS and have developed recurrence cancer could be benefited by WFA treatment alone or in combination with CIS.

## Conclusions

The silent observation from this study is that treatment of mice bearing human ovarian tumors with CIS results in an unwanted expansion of cells that express CSC markers, what may lead to CIS resistance and recurrence of ovarian tumor. In contrast, WFA if employed alone or in combination with CIS ameliorates this unwanted effect. The data obtained from our study suggest that WFA alone or in combination with CIS may serve as a safer and more efficacious therapy for both first line and second line options for ovarian cancer.
